# *Epichloë* Endophytes Shape the Foliar Endophytic Fungal Microbiome and Alter the Auxin and Salicylic Acid Phytohormone Levels in Two Meadow Fescue Cultivars

**DOI:** 10.3390/jof9010090

**Published:** 2023-01-06

**Authors:** Suni Anie Mathew, Marjo Helander, Kari Saikkonen, Radomira Vankova, Petre I. Dobrev, Serdar Dirihan, Benjamin Fuchs

**Affiliations:** 1 Department of Biology, University of Turku, 20014 Turku, Finland; 2 Biodiversity Unit, University of Turku, 20014 Turku, Finland; 3 Laboratory of Hormonal Regulations in Plants, Institute of Experimental Botany of the Czech Academy of Sciences, Rozvojova 263, 16502 Prague, Czech Republic

**Keywords:** grass endophyte, *Festuca*, symbiosis, microbiota, plant hormone, defense response, plant–fungal interactions, holobiont

## Abstract

Plants harbor a large diversity of endophytic microbes. Meadow fescue (*Festuca pratensis*) is a cool-season grass known for its symbiotic relationship with the systemic and vertically—via seeds—transmitted fungal endophyte *Epichloë uncinata,* yet its effects on plant hormones and the microbial community is largely unexplored. Here, we sequenced the endophytic bacterial and fungal communities in the leaves and roots, analyzing phytohormone concentrations and plant performance parameters in *Epichloë*-symbiotic (E+) and *Epichloë*-free (E-) individuals of two meadow fescue cultivars. The endophytic microbial community differed between leaf and root tissues independent of *Epichloë* symbiosis, while the fungal community was different in the leaves of *Epichloë*-symbiotic and *Epichloë*-free plants in both cultivars. At the same time, *Epichloë* symbiosis decreased salicylic acid and increased auxin concentrations in leaves. *Epichloë*-symbiotic plants showed higher biomass and higher seed mass at the end of the season. Our results demonstrate that *Epichloë* symbiosis alters the leaf fungal microbiota, which coincides with changes in phytohormone concentrations, indicating that *Epichloë* endophytes affect both plant immune responses and other fungal endophytes. Whether the effect of *Epichloë* endophytes on other fungal endophytes is connected to changes in phytohormone concentrations remains to be elucidated.

## 1. Introduction

Microbes are essential organismal partners that inhabit every living being on the earth, contributing to the vital life-sustaining functions of their host organism [1,2]. Host-associated microbes interact not only with their host, but also with coexisting microbes, including bacteria, fungi, and viruses [3,4], thus forming a complex “microbial community.” The importance of the microbiome constituting the genetic component of these microbial communities and their metabolic activities has been widely acknowledged. Current sustainable approaches to improve the fitness and health of host plants and animals comprise modifying the parameters of their microbial partners [5,6]. 

Plants are increasingly studied for their microbiome and associated improvement of performance and fitness, particularly under extreme events driven by climate change [7]. The plant holobiont theory defines plants and their microbiome as one entity, and here, plant performance and fitness can be enhanced by association with certain microbial partners [8,9,10]. The plant microbiome can be composed of microbes residing on plant surfaces (epiphytes) or inside tissues (endophytes). Endophytes can be systemic microbes that exist in the plant seed and are vertically transmitted in the host plants. Nonsystemic endophytes, such as soil-borne microbes, are introduced horizontally via rain, wind, or animals from the environment, and thus virtually every biotic and abiotic factor can interact with the plant modifying its microbiome [7,11,12]. These microbes can range from mutualistic and commensalistic to parasitic, with plant-beneficial microbes promoting plant growth, nutrition, and stress resistance [13,14].

Plants respond to interacting microbes often through immune responses. Resistance to pathogenic microbes depends on plant immune responses, which can be boosted and primed by plant-associated beneficial microbes [15,16,17,18]. Most studied examples of microbes improving plant performance are root-associated bacteria in legumes (nitrogen fixing), endo- and ectomycorrhizal fungi, and systemically growing endophytic fungi in grasses. The interactions of these microbes with the plant immune system have been increasingly uncovered. Plant hormones are considered key players in shaping the plant microbiome [19]. Plant immune responses are often mediated by the induction of phytohormones, which can be affected by the presence of microbes or, in turn, regulate the growth and metabolism of endophytic microbes [20]. In general, plants respond to biotrophic microbes with an induction of salicylic acid, and necrotrophic microbes induce jasmonic acid and associated defense pathways [21]. However, an increasing number of studies have confirmed the involvement of other phytohormones and a crosstalk of hormones in plant responses to microbial partners, some of which are capable of producing phytohormones themselves, hence enabling the symbiotic nature between plant and microbe [22,23,24,25,26]. 

Systemic *Epichloë* endophytes are a model organism for studying plant symbiotic microbes [27,28,29]. These fungi are obligate endosymbionts of various cool-season grasses and are transmitted vertically via plant seeds. Asexual *Epichloë* endophytes have been shown to benefit the host plant in high nutrient ecosystems by improving drought tolerance, herbivore resistance, and pathogen resistance [30]. The beneficial properties have been attributed to *Epichloë*-conferred alkaloids, but recent studies have aimed to unravel the role of plant hormones in growth and defense promoted by *Epichloë* endophytes [31,32,33]. Although some studies suggested that *Epichloë* endophytes protect the plant against fungal intruders, such as the ergot fungus in plant seeds, others demonstrated an increased seed infection with ergot fungi on *Epichloë*-symbiotic plants [34,35]. Furthermore, it has been shown that *Epichloë*-symbiotic grass seeds show a higher endophytic bacterial diversity compared with *Epichloë*-free seeds, hence demonstrating their ability to interact with other endophytic microbes [36]. In leaf tissue, on the other hand, *Epichloë* endophytes have shown different trends in shaping microbial communities. *Epichloë* in perennial ryegrass (*Lolium perenne*) did not significantly affect fungal community structure [37], but *Epichloë* in tall fescue (*Festuca arundinaceae* = *Schedonorus phoenix)* altered the community of fungal endophytes, but not the bacterial endophytes, in leaves of the host plants [38]. In another study, the foliar fungal communities in *S.phoenix* were not different between *Epichloë*-symbiotic and *Epichloë*-free plants, but the fungal community structures between the two consecutive sampling years differed [39]. In *Festuca rubra*, the presence of *Epichloë festuca* along with the habitat of the host plant affected the infection frequencies of nonsystemic fungal endophytes [40]. Studies from the grass species *Achnatherum inebrians* showed that the presence of the symbiotic fungal partner *Epichloë gansuensis* reduced the diversity of the root-associated bacterial community [41] while increasing the diversity of endophytic and epiphytic bacterial and fungal phyllosphere communities. It was also found that *Epichloë* did not impact the microbial community structure [42]. However, it remains largely unknown how an *Epichloë* endophyte affects the leaf and root microbiome and whether altered microbiomes can be linked to the hormonal and performance attributes of its host plant.

In the present study, we analyzed whether *Epichloë* endophyte symbiosis can alter the endophytic microbiota of the above- and belowground parts of two meadow fescue (*Festuca pratensis*) cultivars (‘Valtteri’ and ‘Kasper’) and whether the changes would coincide with altered plant performance and phytohormone concentrations. The endophytic bacterial and fungal community compositions of leaves and root tissues in the *Epichloë* endophyte symbiotic (E+) and nonsymbiotic (E-) plants were determined using targeted sequencing of the 16S rRNA gene and ITS (internal transcribed sequence) regions. Additionally, plant performance and phytohormone concentrations of the E+ and E- host cultivars were studied to understand their role in shaping the structure and composition of endophytic microbial communities associated with *Epichloë*. We hypothesize that *Epichloë* endophytes (1) shape the endophytic microbial communities particularly aboveground, (2) which coincides with changes in phytohormone concentrations, and (3) improves plant performance.

## 2. Materials and Methods

### 2.1. Plant Material and Study Setup

We chose two meadow fescue (*Festuca pratensis* L.) cultivars (‘Kasper’, and ‘Valtteri’) as our model because meadow fescue is widely used as pasture and forage grass in Europe and commonly harbors the systemic seed transmitted fungal endophyte *Epichloë uncinata* [(W. Gams, Petrini and D. Schmidt) Leuchtm. and Schardl.]. We obtained the seeds of the cultivars from seed producers via the Finnish Food Authority (www.ruokavirasto.fi, accessed on 15 March 2017). Through staining and microscopy of 100 seeds, we verified that all the seed lots of the cultivars had both *Epichloë*-free (E-) and *Epichloë*-symbiotic (E+) seeds [43]. The endophyte frequency in the seed lots varied between 30% and 90%. We verified the final endophyte status (E- or E+) of each experimental plant in the field using the results from ITS-targeted polymerase chain reaction (PCR) for fungal community analysis. Because of the mortality of the plants, at the end of the experiment, we had 7 E- and 5 E+ ‘Kasper’ plants and 5 E- and 5 E+ ‘Valtteri’ plants. 

We first grew plants in pots (May 2017) in the greenhouse and then transferred 20 plants from each cultivar to the experimental field in the Turku University Botanical Garden (60°26′ N, 22°10.4′ E) (June 2017) in a randomized design. The plants were planted 0.5 m apart from each other, hand-weeded to avoid competition from other plant species, and watered if needed during the first growing season. The field was fenced with a metal net to keep out mammalian herbivores. The soil in the experimental area was ~90% clay with some sand and peat. The soil pH was 6.2, and content of phosphorus 4.2 mg/L, potassium 250 mg/L, calcium 1900 mg/L, magnesium 570 mg/L, sulfur 10.6 mg/L, zinc 2.74 mg/L, copper 7.5 mg/L, and manganese 15 mg/L. We did not use fertilizers or pesticides in the area. The average temperature (Jun–-Aug) was 17 °C, and the average relative humidity was 70% at the field site during the growing season (data accessed on 15 December 2022 from Finnish Meteorological Institute; www.ilmatieteenlaitos.fi).

### 2.2. Plant Performance Parameters

We recorded the growth, reproduction, and chlorophyll content (SPAD) of the experimental plants at the beginning of August 2019. To estimate the growth, we measured the average height of the plant, height of the longest leaf, circumference of the tuft at 5 cm above the ground, and number of tillers. For the reproduction estimate, we counted the number of flower heads, and for the chlorophyll content, we measured the SPAD values (Minolta SPAD-502 Plus meter) from three randomly chosen leaves per experimental plant.

### 2.3. Sample Collection

We collected plant samples for phytohormone and microbiome analysis from the experimental field in mid-August 2019. For phytohormone analysis, we sampled two to three healthy leaves from each plant, which were weighed and immediately stored in liquid nitrogen until further processing.

We sampled two to three healthy leaves and approximately 100 mg of root from five to seven replicates each of E+ and E- ‘Valtteri’ and ‘Kasper’ cultivars in sterile plastic bags on ice. In the laboratory, the samples were washed with tap water, dried, and weighed to ensure 100 mg samples. The samples were then surface sterilized in a laminar air flow hood using 70% ethanol (1 min), followed by 3% sodium hypochlorite solution (3 min), rinsed thrice with sterile distilled water (3 × 1 min), and air-dried. The samples were transferred to 2 mL microcentrifuge tubes and stored at −80 °C until further processing.

### 2.4. Microbiome Analysis 

The frozen samples were homogenized using a bead mill homogenizer (Bead Ruptor 96-Well Plate Homogenizer, Kennesaw, GA, OMNI International US), and DNA was extracted using an Invisorb Spin Plant Mini Kit (STRATEC Biomedical AG, Birkenfeld, Berlin, Germany). Following DNA extraction, the 16S rRNA gene and ITS regions from the DNA samples were amplified by PCR.

From the DNA samples, the variable regions V6-V8 of bacterial 16S rRNA genes were amplified by a nested PCR approach. We performed the first round of PCR reactions with 30 ng DNA, 1X PCR buffer, 0.2 mM dNTPs, 0.3 μM each of primers 799F (AACMGGATTAGATACCCKG) [44], and 1492R (GGYTACCTTGTTACGACTT) (modified from [45]) and 2000 U/mL GoTaq DNA Polymerase (Promega, Madison, WI, USA) in a 30 μL reaction volume. This was followed by a second round of PCR with 1:10 diluted PCR product from round 1, 1X PCR buffer, 0.2 mM dNTPs, 0.3 μM and M13-1062F (TGTAAACGACGGCCAGTGTCAGCTCGTGYYGTGA, [46,47] and 1390R (ACGGGCGGTGTGTRCAA) [48] primers. A third round of PCR was done with 1:1 dilution of the second PCR product, IonA-barcode-M13 primers [47] for sample tagging, and 1390R and1390R-P1 for IonTorrent PGM sequencing. The PCR profile was as follows: 3 min initial denaturation at 95 °C, denaturing at 95 °C for 45 s, annealing at 54 °C for 45 s, and extension at 72 °C for 1 min, and final extension at 72 °C for 5 min was set on a thermocycler (BIORAD). The same protocol was followed with 35 cycles for first round, and 25 and 8 cycles for the second and third rounds, respectively. 

The ITS regions were amplified using primers M13-ITS7F (TGTAAAACGACGGCCAGTGTGARTCATCGAATCTTTG) and ITS4R (TCC TCC GCT TAT TGA TAT GC) [49]. The 30 µL reaction mixture contained 30 ng of sample DNA, 1x PCR buffer, 0.2 mM dNTPs, 0.3 μM of each primer, and 1250 U/mL GoTaq DNA Polymerase (Promega, Madison, WI, USA). We performed the second round of PCR using 1:10 dilution of the first PCR product as a template, 1x PCR buffer, 0.2 mM dNTPs, 0.3 μM of each primer, and 1250 U/mL GoTaq DNA Polymerase, barcode-M13 forward primer, and ITS4-P1 (CCTCTCTATGGGCAGTCGGTGATTCCTCGCTTATTGATATGC) as the reverse primer. The amplification profile was 5 mins initial denaturation at 95 °C, followed by denaturing, annealing, and extension at 95 °C for 30 s, 55 °C for 30 s, and 72 °C for 1 min, repeated at 35 cycles for the first round and 8 cycles for the second round. The final extension was carried out at 72 °C for 7 mins. 

The PCR products were run on 1.5% agarose gel and those without good quality DNA and low amplification were not processed further. Three to seven replicates from each sample were quantified on an Agilent 2100 Bioanalyzer system, pooled to obtain a sequence library, size-fractionated on 2% agarose gel cassette (Marker B) using Pippin Prep (Sage Science, Beverly, MA, USA), and sequenced on Ion 314™ Chip v2 in Ion Personal Genome Machine™ (ThermoFisher Scientific Ltd., Waltham, MA, USA).

### 2.5. Plant Hormone Extraction and Quantification

We analyzed plant hormones, as described in Dobrev and Vankova (2012) [50] and Fuchs et al. (2022) [51]. In brief, approximately 100 mg of fresh plant material was homogenized under constant liquid nitrogen supply, which was followed by an extraction with cold (−20 °C) methanol/water/formic acid (15/4/1, v/v/v) in a 2 mL reaction tube (Eppendorf GmbH). The following isotope-labeled internal standards (10 pmol per sample) were added: ^13^C_6_IAA and ^2^H_4_-OxIAA (Cambridge Isotope Laboratories, Tewksbury, MA, USA); ^2^H_4_-SA [Sigma-Aldrich, St. Louis, MO, USA); ^2^H_3_-PA, ^2^H_3_-DPA, ^2^H_4_-7OH-ABA, and ^2^H_5_-ABAGE (NRC-PBI); and ^2^H_6_-ABA and ^2^H_5_-JA, before subsequent centrifugation (17,000× *g*, 4 °C, 20 min). The extract was centrifuged (17,000 *g*, 4 °C, 20 min) to remove solid debris. It was then concentrated using an Alpha RVC vacuum centrifuge (Christ; 40 °C, 15 mbar, 1.5 h). Phytohormones were purified using a reverse-phase–cation exchange solid-phase extraction (SPE) column (Oasis-MCX, Waters) and eluted with methanol, concentrated to dryness, and resuspended in 30 µL acetonitrile (15%). Hormones were analyzed on an HPLC (Ultimate 3000, Dionex) coupled to a 3200 Q TRAP hybrid triple quadrupole/linear ion trap mass spectrometer (Applied Biosystems) and quantified by an isotope dilution method with multilevel calibration curves (r^2^ > 0.99). Data were processed with the Analyst 1.5 software package (Applied Biosystems). The analyses covered the plant hormones abscisic acid (ABA), indole acetic acid (IAA), phenyl acetic acid (PAA), salicylic acid (SA), and jasmonic acid (JA). Furthermore, we quantified a potential SA precursor, benzoic acid (BzA), and the main product in ABA metabolism phaseic acid (PA). 

### 2.6. Bioinformatics and Statistical Analys

The sequence reads from the microbiome analysis were processed using CLC Genomics Workbench 11.0 with a Microbial Genomics Module (https://digitalinsights.qiagen.com/products-overview/discovery-insights-portfolio/analysis-and-visualization/qiagen-clc-microbial-genomics-module/ accessed on 10 October 2020). After filtering low-quality and <150 bp sequence reads, high-quality reads were aligned and clustered into OTUs (Operational Taxonomic Units) at 97% sequence identity. The OTUs were taxonomically classified using reference databases RDP 16S rRNA training set 16 for bacteria and UNITE Fungal ITS trainset 7.1 for fungi (https://rdp.cme.msu.edu accessed on 1 December 2020) [52]. OTUs representing plant genes with less than a total count of 10 reads were eliminated. Rarefaction curves were constructed in Paleontological Statistics (PAST) software version 4.12 (https://www.nhm.uio.no/english/research/resources/past/ accessed on 27 December 2022) for each individual sample, showing the number of observed OTUs to the number of acquired reads. Statistical analyses were conducted separately for bacterial and fungal communities using PRIMER-7 software with PERMANOVA+ add-on (https://www.primer-e.com/ accessed on 6 January 2021). Permutational multivariate analysis of variance (PERMANOVA) tests and principal co-ordinate analysis (PCoA) to find the effect of *Epichloë* on the overall microbial structure community were performed on Bray–Curtis distance matrices of square root transformed abundance data. With the similarity percentages–species contribution (SIMPER) analysis, we identified OTUs or species majorly contributing to the differences between community structures. The statistical analyses were repeated on fungal datasets after removing OTUs assigned as *Epichloë*.

Plant performance parameters showed normal distribution and were analyzed with a Student’s *t*-test. Phytohormone concentrations were not normally distributed and analyzed with the generalized linear model (GLM). A graphical illustration was done with the ggplot2 package in the software R.

## 3. Results

### 3.1. Epichloë Shapes Endophytic Fungal Community Composition in Leaves and Not in Roots 

**Figure 1:**
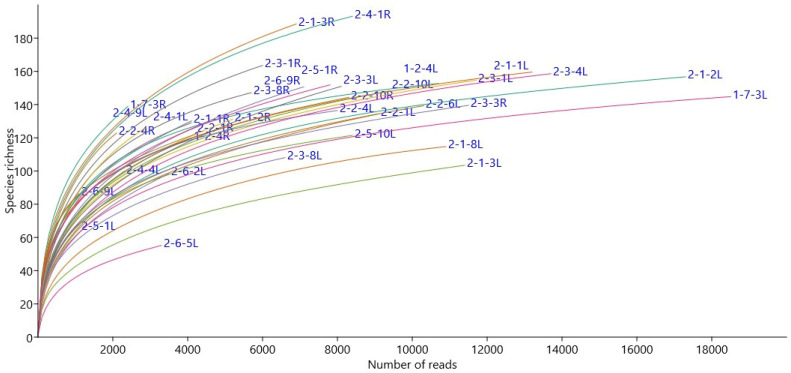
Rarefaction curves for each sample for endophytic fungal community data in meadow fescue (*Festuca pratensis*) showing species richness to the number of reads acquired. Each curve is marked with the sample name and specific tissue (L: leaf, R: root).

**Figure 2:**
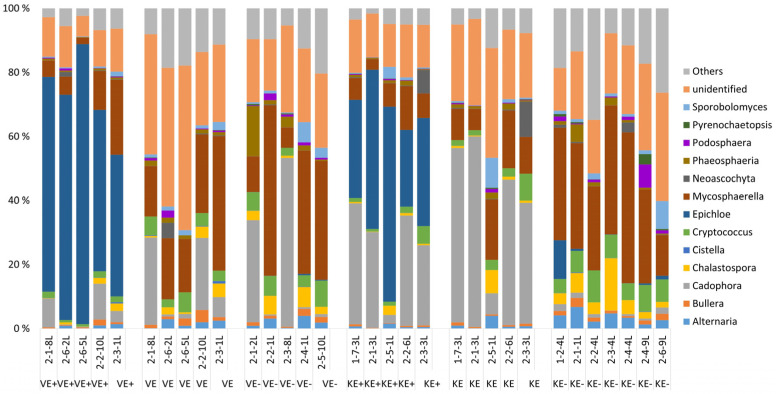
Taxonomic distribution of fungal communities at the genus level in leaves of *Epichloë*-symbiotic (E+) and *Epichloë*-free (E-) plants of ‘Valtteri’ (V) and ‘Kasper’ (’K) cultivars of meadow fescue (*Festuca pratensis*). For each cultivar, the *X*-axis shows taxonomic distribution in E+ plants for total fungal communities (E+), fungal communities in E+ plants after depletion of *Epichloë* taxa (E) and fungal communities in E- plants (E-).

A total of 276,216 good quality fungal sequence reads were obtained and classified into 438 OTUs representing three phyla (Ascomycota, Basidiomycota, and Glomeromycota) and belonging to 75 families and 103 genera. The rarefaction curve for each sample for fungal microbial community data is given (Figure 1). The genus *Epichloë* dominated 65% and 39% of the relative abundance of the fungal communities in leaves of *Epichloë*-symbiotic (E+) plants of the ‘Valtteri’ and ‘Kasper’ cultivars, respectively (Figure 2).

**Figure 3:**
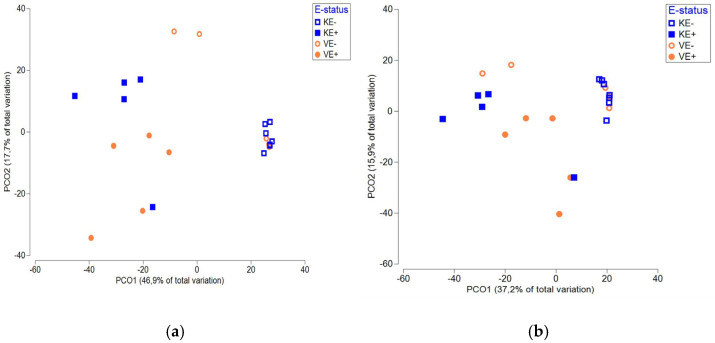
PCoA analysis on (**a**) total endophytic fungal communities and (**b**) *Epichloë*-depleted fungal communities in ‘Kasper’ *Epichloë*-symbiotic (KE+), ‘Kasper’ *Epichloë*-free (KE-), ‘Valtteri’ *Epichloë*-symbiotic (VE+) and ‘Valtteri’ *Epichloë*-free (VE-) leaves of meadow fescue (Festuca pratensis) as indicated by the *Epichloë*-status (E-status) in the figure.

The next major genera were *Mycosphaerella* and *Cadophora*. For *Mycosphaerella*, the relative abundance was lower in E+ plants (‘Valtteri’ 13%; ‘Kasper’ 8%) and higher in E- plants for both cultivars (‘Valtteri’ 31%; ‘Kasper’ 47%). The relative abundance of *Cadophora* was lower in E+ plants compared with E- plants in ‘Valtteri’ (E+ leaves 5%; E- leaves 20%), while in E+ ‘Kasper’ plants, their relative abundance was higher (E+ leaves 31%; E- leaves 0.02%) (Figure 1). The presence of *Epichloë* clearly impacted the composition of the endophytic fungal communities in leaves for both ‘Valtteri’ and ‘Kasper’ cultivars, as evidenced by PERMANOVA analysis (Table 1) and visualized by PCoA (Figure 3a). The PERMANOVA and PCoA analysis on *Epichloë*-depleted datasets was performed to ensure that the fungal community structures were significantly different in the E+ and E- leaves of both cultivars (Table 2, Figure 3b). 

With the SIMPER analysis on leaves, the major species that contributed to dissimilarity between E+ and E- plants in both cultivars were *Epichloë*, *Mycosphaerella tassiana*, *Cadophora,* and Heliotales spp. In the *Epichloë*-depleted datasets, in addition to the above taxa other than *Epichloë*, the relative abundances of *Phaeosphaeria triglochinicola* and Pleosporales sps. were relatively lower in VE+ plants. Another interesting observation was that the relative abundance of Pleosporales sps. was higher in VE+ plants compared with VE- plants, but in contrast, the relative abundance of Pleosporales sps. was lower in the KE+ plants. Furthermore, the relative abundance of *Vishniacozyma victoriae* was remarkably lower in the KE+ leaves (Table 3). A detailed table on the SIMPER analysis is provided in Table S1 in the Supplementary Material.

**Figure 4:**
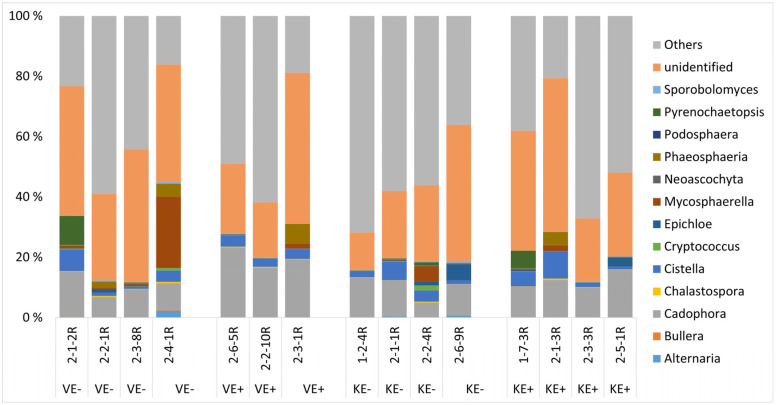
Taxonomic distribution of fungal communities at the genus level in the *Epichloë*-symbiotic (E+) and *Epichloë*-free (E-) roots of ‘Valtteri’ (V) and ‘Kasper’ (K) cultivars of meadow fescue (*Festuca pratensis*).

For the roots, *Epichloë* status did not impact the overall structure of the fungal communities. The taxonomic distribution of the fungal communities in the roots (Figure 4) showed major genera, including *Cadophora*, *Ophiosphaerella*, *Phaeosphaeria*, *Rhexocercosporidium,* and *Scytalidium,* in both cultivars of the E+ and E- plants. We also compared E+ leaves of ‘Valtteri’ and ‘Kasper’ and E- plants of both cultivars to analyze whether cultivar-specific changes occurred in the communities, but found no significant differences.

### 3.2. Epichloë Does Not Impact Endophytic Bacterial Community Structure

**Figure 5:**
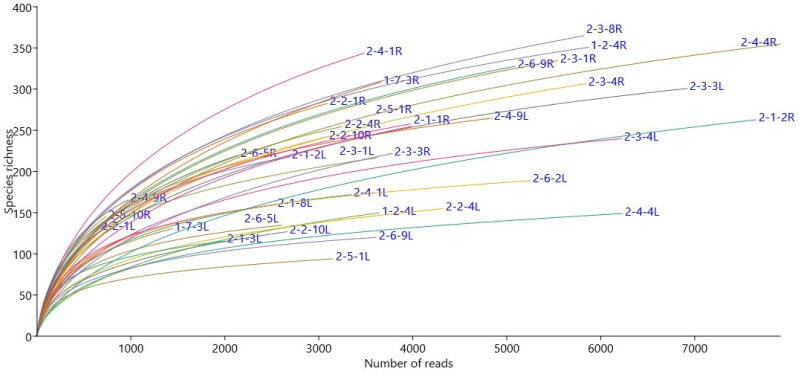
Rarefaction curves for each sample for endophytic bacterial community data in meadow fescue (*Festuca pratensis*) showing species richness to the number of reads acquired. Each curve is marked with the sample name and specific tissue (L: leaf, R: root).

**Figure 6:**
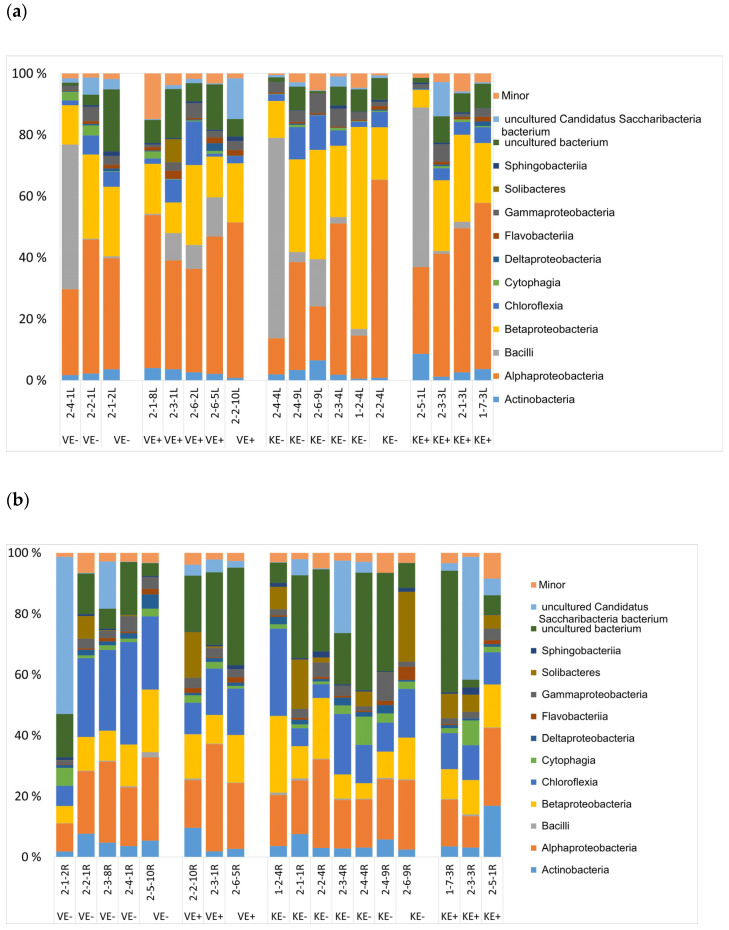
Taxonomic distribution of bacterial communities at the phyla level in the (**a**) leaves and (**b**) roots of ‘Valtteri’ (V) and ‘Kasper’ (K) cultivars of meadow fescue (*Festuca pratensis*).

**Figure 7:**
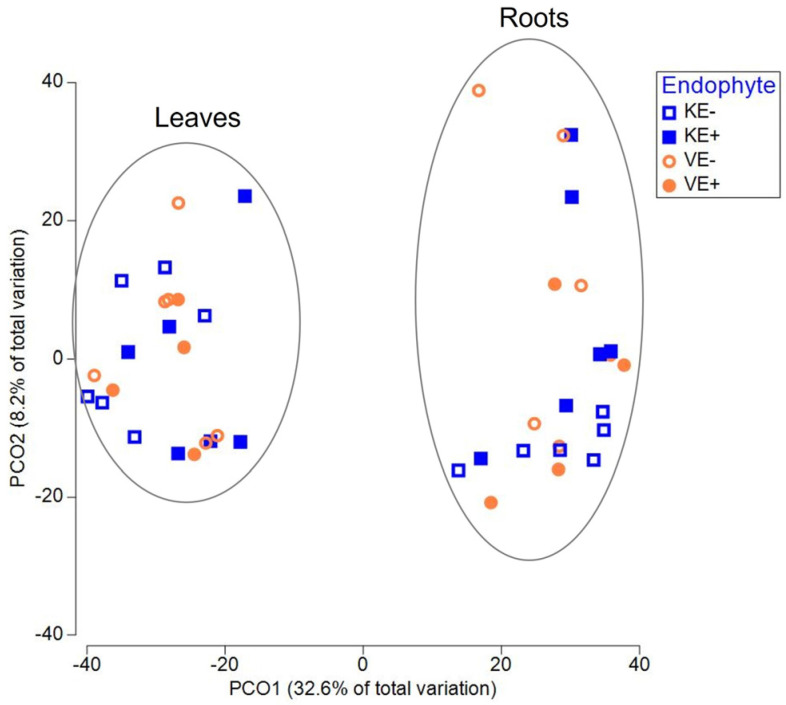
PCoA analysis of the bacterial communities in the leaves and roots of meadow fescue (*Festuca pratensis*) cultivars ‘Kasper’ and ‘Valtteri’. ‘Kasper’ *Epichloë*-free plants (KE-), ‘Kasper’ *Epichloë*-symbiotic plants (KE+), ‘Valtteri’ *Epichloë*-free plants (VE-), ‘Valtteri’ *Epichloë*-symbiotic plants (VE+).

The bacterial community structure and rarefaction curves (Figure 5) were deciphered from 153,824 final sequence reads divided into 755 OTUs. The community structure consisted of 12 phyla, as represented by 110 families and 212 genera. The major genera in the leaves were *Sphingomonas*, *Hymenobacter*, *Massilia*, and *Methylobacterium* and in the roots were *Roseiflexus*, *Shinella*, *Rhizobium,* and *Rhizobacter*. The taxonomic distribution of bacterial communities in the leaves (Figure 6a) and roots (Figure 6b) of the two cultivars (‘Valtteri’ and ‘Kasper’) was not different between the E+ and E- plants. The PERMANOVA (Supplementary material Table S2) and PCoA analysis (Figure 7) showed there were no significant differences in the bacterial community structures of E+ and E- plants, indicating *Epichloë* does not impact endophytic bacterial community structure in leaves or roots.

### 3.3. Epichloë Symbiosis Alters Plant Parameters

**Figure 8:**
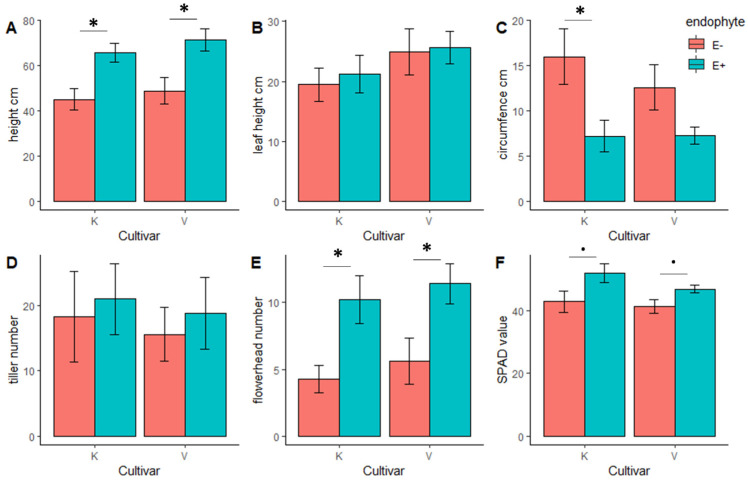
Plant parameters of meadow fescue (*Festuca pratensis*) cultivars ‘Kasper’ (K) and ‘Valtteri’ (V) were compared between *Epichloë*-symbiotic (E+) and *Epichloë*-free (E-) plants. Data for (**A**): plant height (cm), (**B**): leaf length (cm), (**C**): circumference (cm), (**D**): tiller number, (**E**): number of flowerheads, and (**F**): SPAD value were analyzed with a Student’s *t*-test for both cultivars separately. N = 5–6. For significance levels, see Table 4. * = *p* < 0.05; . = *p* < 0.1.

The *Epichloë* endophyte (E+)-symbiotic plants were taller and had a higher number of flower heads compared with their nonsymbiotic (E-) counterparts in both cultivars (Table 4, Figure 8). In contrast, plant circumference was smaller in the E+ plants of the cultivar ‘Kasper’ showing a trend to be smaller in the E+ ‘Valtteri’ cultivar (Table 4, Figure 8). Chlorophyll content was measured as SPAD value and by trend higher in E+ plants compared with E- plants (Table 4, Figure 8).

### 3.4. Epichloë Symbiosis Alters Plant Hormone Concentrations

**Figure 9:**
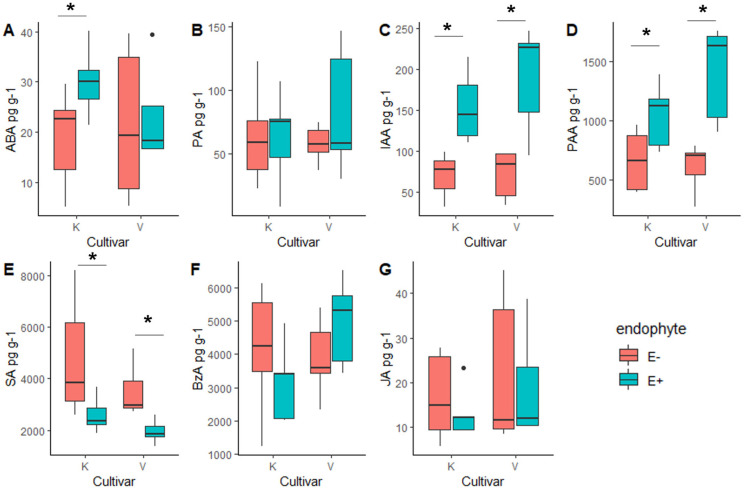
Phytohormone concentrations of meadow fescue (*Festuca pratensis*) cultivars ‘Kasper’ (K) and ‘Valtteri’ (V) were compared between *Epichloë*-symbiotic (E+) and *Epichloë*-free (E-) plants (GLM). Median and 95% confidence intervals are shown. The analyses covered the plant hormones (**A**): abscisic acid (ABA), (**B**): the main product in ABA metabolism phaseic acid (PA), (**C**): indole acetic acid (IAA), (**D**): phenyl acetic acid (PAA), (**E**): salicylic acid (SA), (**F**); a potential SA precursor benzoic acid (BzA) and (**G**): jasmonic acid (JA). Dots show outliers beyond 1.5-times the interquartile range, which is represented by whiskers; * = *p* < 0.05.

Plants symbiotic to *Epichloë* showed significantly higher auxin concentrations (IAA and PAA) compared with nonsymbiotic plants in both cultivars (Table 5, Figure 9). Furthermore, ABA increased in *Epichloë*-symbiotic plants from the Kasper cultivar. In contrast, the phytohormone SA was lower in the *Epichloë*-symbiotic plants from both cultivars (Table 5, Figure 9).

## 4. Discussion

In the present study, *Epichloë* symbiosis (E+) in the host plant meadow fescue, *F. pratensis,* clearly impacted the structure of the endophytic fungal community in leaves, but not in roots. This confirms our hypothesis that *Epichloë* endophyte is one of the major factors shaping the endophytic fungal communities in the aboveground parts of its host plants [38]. *Epichloë* was relatively the most abundant genus in the leaves of E+ plants of both cultivars ‘Valtteri’ and ‘Kasper’. The endophytic fungal community structures of E+ leaves differed considerably from the *Epichloë*-uninfected (E-) leaves. *Epichloë* symbiosis did not alter the structure of the endophytic bacterial community in the leaves or roots of the host plant. Finally, E+ plants were taller, with more flower heads in both cultivars. Both cultivars showed higher auxin and lower SA concentrations in E+ plants, and ABA concentrations were significantly higher in E+ plants than in E- plants of the ‘Kasper’ cultivar. Our study is the first to examine both the endophytic microbial communities and phytohormone concentrations, while the mechanistic link between both needs to be unraveled. 

*Epichloë* endophytes grow in the intercellular space of the leaves, stems, and reproductive tissue of several cool-season grasses and are typically absent in the roots [53,54], indicating that their effect on shaping fungal community structure is limited to aboveground plant parts. This is in agreement with our previous study, where *Epichloë* symbiosis in tall fescue (*Schedonorus phoenix*) impacted only the endophytic fungal community in leaves, not the bacterial community [38]. However, *Epichloë* symbiosis in tall fescue have also been reported not to impact foliar fungal community structures [39]. Significant differences in the structures of fungal communities were observed in the dataset, with and without depleting *Epichloë* taxa and, hence, confirming the role of *Epichloë* in shaping the endophytic mycosphere. The most abundant endophytic fungal genera next to *Epichloë* were *Mycosphaerella* and *Cadophora*. *Epichloë* symbiosis in *Lolium perenne* similarly impacted its foliar fungal composition, with *Mycosphaerella* being the second-most dominant fungi apart from *Epichloë* [37]. In general, the impact of *Epichloë* symbiosis on the foliar fungal community was more pronounced in the ‘Kasper’ cultivars compared with ‘Valtteri’ ones.

The relative abundance of *Epichloë* OTUs belonging to *Epichloë uncinata*, here specifically forming symbiotic relations with meadow fescue [55], slightly differed between the cultivars. *Epichloë* symbiosis is common, but frequencies of infection can vary among cultivars, depending on the infection status of the mother plants and genetic and biotic and abiotic environment [56,57,58,59]. The noticeable pattern of foliar endophytic fungal assemblage observed in the ‘Kasper’ cultivar reflects the complexity of the relation of mutualistic endophyte *Epichloë* with its specific host cultivar. From the SIMPER analysis of the fungal community in leaves, the relative abundance of *Mycosphaerella tassiana* was lower in the E+ plants compared with the E- plants in both varieties, but more prominent in ‘Kasper’. *M. tassiana* is a commonly known plant pathogen in wheat [60] and other grass species [61]. Our results have indicated that *Epichloë* symbiosis may be an advantage, keeping pathogen such as *M. tassiana* levels inactive. Several species of *Epichloë* restrict or inhibit plant pathogens through fungi–fungi interactions, such as producing fungistatic chemicals, competing for favorable niches, or eliciting changes in the host plant endosphere [62,63,64].

The relative abundance of *Cadophora* was slightly higher in uninfected ‘Valtteri’ leaves, but significantly lower in uninfected ‘Kasper’ leaves. The complex cultivar-specific compatibility of each cultivar with *Epichloë* may explain this contrasting trend of relative abundance of *Cadophora*, which is considered a pathogenic fungus. The relative abundance of another microbial species, *Vishniacozyma victoriae,* was higher in the nonsymbiotic leaves of ‘Kasper’. *Epichloë*-symbiotic plants generally have more fitness than their nonsymbiotic counterparts, which then harbor beneficial microbes, such as *V. victoriae* because it is one of the cold-adapted endophytic fungi producing various bioactive and antimicrobial metabolites, enzymes, and hormones helping in plant growth and ecological adaptation to cold environments [65].

*Epichloë*-symbiotic plants showed higher plant performance with increased plant height, number of flower heads, and higher chlorophyll content. Similar to our study, better plant growth of *Epichloë*-symbiotic grasses has been commonly documented and may be attributed to increased concentrations of auxins, which are common growth-promoting phytohormones throughout the plant kingdom and are connected to physiological changes in endophyte-symbiotic grasses [66]. The inducing effect of endophyte symbiosis on SA concentrations has been connected to a better defense response against biotrophic pathogens and piercing sucking insects [32,67]. On the other hand, SA reduction in symbiotic plants has been reported earlier in *Epichloë*-symbiotic plants and can be an *Epichloë*-mediated suppression of the host immune response for a better intercellular establishment [68]. Because of the key role of SA in interactions with biotrophic pathogens, the observed reduction in SA concentrations in endophyte-symbiotic plants is likely to be responsible for changes in the endophytic mycosphere [31]. On the other hand, the *Epichloë* symbiosis may first alter the endophytic microbial community, hence leading to altered plant responses at the hormonal level [18]. Hence, *Epichloë* symbiosis shapes the endophytic fungal community, and because of this tripartite symbiont–host plant–endophyte interaction, they mutually coexist and coevolve.

## 5. Conclusions

In conclusion, our results have demonstrated the central role of systemic *Epichloë* endophytes in cool-season grasses affecting the community of the fungal leaf endophytic microbiota and indicating a link to plant hormone concentrations related to resistance against biotrophic pathogens. The endophyte features of improving plant resistance, biomass, and reproduction are increasingly unraveled to be linked to multiple transcription factors and hormone regulation. It remains to be elucidated whether the effect presented here on the fungal microbiome is caused by direct effects of the *Epichloë* fungi on other fungal species or whether it is indirectly caused by *Epichloë*-mediated changes in the plant physiology, such as phytohormone concentration causes.

## Figures and Tables

**Table 1:** PERMANOVA results for total fungal communities in meadow fescue. Df: degrees of freedom, SS: Sum of squares, MS: mean sum of squares, Pseudo-F: F-value by permutation, P(perm): *p*-values based on more than 900 permutations.

Source	df	SS	MS	Pseudo-F	P(perm)	Unique Perms
Tissue	1	33964	433964	30.86	0.001	998
Endophyte	3	9306.3	3102.1	22.82	0.001	997
Tissue × Endophyte	3	6968.3	2322.8	2.11	0.001	997
PERMANOVA analysis on total fungal communities for leaves						
Endophyte	3	14915	4971.8	66.1988	0.001	998
PERMANOVA analysis on total fungal communities for roots						
Endophyte	3	4130	1376.7	0.840	0.79	997

**Table 2:** PERMANOVA results for *Epichloë*-depleted fungal communities in meadow fescue.

Source	df	SS	MS	Pseudo-F	P(perm)	Unique Perms
Tissue	1	34398	34398	27.47	0.001	998
Endophyte	3	7297.7	2432.6	1.9426	0.001	995
Tissue × Endophyte	3	5175.1	1725	1.3776	0.028	998

**Table 3:** SIMPER (similarity percentages) analysis in the leaves of E+ and E- plants in total and *Epichloë*-depleted fungal communities (VE-: *Epichloë*-free ‘Valtteri’ plants, VE+: *Epichloë*-symbiotic ‘Valtteri’ plants, KE-: *Epichloë*-free ‘Kasper’ plants, and KE+: *Epichloë*-symbiotic ‘Kasper’ plants) showing microbial taxa with difference in average relative abundances (Av.Abund: average abundance).

**Total fungal communities**			
**VE-**	**VE+**		
**Av.Abund**	**Av.Abund**	**Taxa**	
0.02	59.43	*Epichloë*	
28.53	8.75	*Mycosphaerella_tassiana*	
**KE-**	**KE+**		
**Av.Abund**	**Av.Abund**		
0.03	36.83	*Epichloë*	
30.87	7.06	*Mycosphaerella_tassiana*	
0.03	18.91	*Cadophora*	
0.01	7.82	Heliotales_unidentified	
***Epichloë*-depleted communities**			
**VE-**	**VE+**		
**Av.Abund**	**Av.Abund**	**Taxa**	
28.60	19.39	*Mycosphaerella_tassiana*	
12.11	7.81	*Cadophora*	
5.27	3.08	Heliotales_sps.	
5.93	7.75	Pleosporales_sps.	
4.25	0.72	*Phaeosphaeria triglochinicola*	
4.70	2.01	Capnodiales sps.	
**KE-**	**KE+**		
**Av.Abund**	**Av.Abund**		
0.03	29.32	*Cadophora*	
31.6	11.49	*Mycosphaerella_tassiana*	
0.01	12.1	Heliotales_sps.	
6.48	4.06	Pleosporales_sps.	
7.36	1.83	*Vishniacozyma victoriae*	

**Table 4:** The effect of *Epichloë* symbiosis on the plant parameters in the two meadow fescue cultivars was analyzed with a Student’s *t*-test. Significant *p*-values are highlighted in bold. N = 5–6.

	‘Kasper’			‘Valtteri’		
	t	F	*p*-Value	t	F	*p*-Value
Plant height	−3.172	9.939	**0.01**	−2.937	7.793	**0.02**
Longest leaf	−4.23	8.993	0.68	−0.148	7.203	0.89
Circumference	2.474	9.019	**0.04**	1.961	5.119	0.11
Tiller no.	−0.308	9.981	0.76	−0.468	7.387	0.653
Flower head no.	−2.895	6.592	**0.02**	−2.539	7.859	**0.04**
SPAD value	−1.982	9.951	0.08	−2.144	6.163	0.07

**Table 5:** Phytohormone concentrations were compared between *Epichloë*-symbiotic (E+) and *Epichloë*-free (E-) plants (GLM) for both meadow fescue (*Festuca pratensis*) cultivars (‘Kasper’ and ‘Valtteri’). Significant *p*-values are highlighted in bold. The analyses covered the plant hormones abscisic acid (ABA), indole acetic acid (IAA), phenyl acetic acid (PAA), salicylic acid (SA), jasmonic acid (JA), a potential SA precursor benzoic acid (BzA), and the main product in ABA metabolism phaseic acid (PA).

Phytohormone	‘Kasper’		‘Valtteri’	
	F	*p*-Value	F	*p*-Value
ABA	3.32	0.04	0.21	0.84
PA	0.07	0.95	1.06	0.32
IAA	4.2	<0.01	3.68	<0.01
PAA	2.52	0.03	3.92	<0.01
SA	2.43	0.05	3.12	0.01
BzA	1.18	0.27	1.37	0.21
JA	0.78	0.45	0.35	0.74

## Data Availability

The data is available upon reasonable request.

## Supplementary Materials

The following supporting information can be downloaded at: https://www.mdpi.com/article/10.3390/jof9010090/s1. Table S1: SIMPER (similarity percentages) analysis in meadow fescue (*Festuca pratensis*) leaves of E+ and E- plants in total and *Epichloë*-depleted fungal communities (VE- ‘Valtteri’ plants without *Epichloë,* VE+ - ‘Valtteri’ with *Epichloë,* KE- ‘Kasper’ without *Epichloë* and KE+ ‘Kasper’ with *Epichloë*) showing microbial taxa with difference in average relative abundances. OTUs: operational taxonomic units; Av.Abund: average abundance; Av.Diss.: average dissimilarity; Diss/SD: dissimilarity/standard deviation; Contrib%: contribution percentage; Cum%: cumulative percentage. Table S2: PERMANOVA results for bacterial communities in meadow fescue (*Festuca pratensis*); df: degrees of freedom, SS: Sum of squares, MS:Mean sum of squares, Pseudo-F: F-value by permutation, P(perm): *p*-values based on more than 900 permutations.
