# Supplementary material for: Epichloë Endophytes Shape the Foliar Endophytic Fungal Microbiome and Alter the Auxin and Salicylic Acid Phytohormone Levels in Two Meadow Fescue Cultivars
Source: J Fungi (Basel). 2023 Jan 6;9(1):90. doi: 10.3390/jof9010090 (PMC9861471; doi:10.3390/jof9010090)
Supplement: Supplementary file 1 [file jof-09-00090-s001.zip › jof-2082466-supplementary.pdf]

Supplementary Material

Table S1: SIMPER (Similarity Percentages) analysis in meadow fescue (*Festuca pratensis*) leaves of E+ and E- plants in total and *Epichloë*-depleted fungal communities (VE- 'Valtteri' plants without *Epichloë,* VE+ - 'Valtteri' with *Epichloë,* KE- 'Kasper' without *Epichloë* and KE+ 'Kasper' with *Epichloë*) showing microbial taxa with difference in average relative abundances. OTUs – Operational Taxonomic Units, Av.Abund – Average abundance, Av.Diss. – Average dissimilarity, Diss/SD – Dissimilarity/ Standard Deviation, Contrib% - Contibution percentage, Cum% - Cumulative percentage

Total communities

Groups VE- & VE+
Average dissimilarity = 77.97

	Group VE-	Group VE+					
**OTUs**	**Av.Abund**	**Av.Abund**	**Av.Diss**	**Diss/SD**	**Contrib%**	**Cum.%**	**Taxa**
otu2	0.02	59.43	29.7	4.12	38.1	38.1	*Epichloë*
otu1	28.53	8.75	11.24	1.4	14.42	52.52	*Mycosphaerella_tassiana*

Groups KE- & KE+
Average dissimilarity = 81.97

	Group KE-	Group KE+					
**OTUs**	**Av.Abund**	**Av.Abund**	**Av.Diss**	**Diss/SD**	**Contrib%**	**Cum.%**	
otu2	0.03	36.83	18.4	2.88	22.45	22.45	*Epichloë*
otu1	30.87	7.06	11.94	2.24	14.57	37.02	*Mycosphaerella_tassiana*
otu3	0.03	18.91	9.45	1.8	11.52	48.55	*Cadophora*
otu4	0.01	7.82	3.91	1.89	4.77	53.31	Heliotales_unidentified

***Epichloë*-depleted communities**

Groups VE- & VE+
Average dissimilarity = 55.99

	Group VE-	Group VE+					
**OTUs**	**Av.Abund**	**Av.Abund**	**Av.Diss**	**Diss/SD**	**Contrib%**	**Cum.%**	**Taxa**
otu1	28.60	19.39	10.02	1.38	18.49	18.49	*Mycosphaerella_tassiana*
otu3	12.11	7.81	7.16	1.14	13.22	31.71	*Cadophora*
otu4	5.27	3.08	3.09	1.12	5.70	37.42	Heliotales_sps.
otu5	5.93	7.75	2.89	1.33	5.34	42.75	Pleosporales_sps.
otu12	4.25	0.72	1.97	0.66	3.65	46.40	*Phaeosphaeria triglochinicola*
otu11	4.70	2.01	1.96	0.97	3.61	50.01	Capnodiales sps.

Groups KE- & KE+
Average dissimilarity = 72.32

	Group KE-	Group KE+					
**OTUs**	**Av.Abund**	**Av.Abund**	**Av.Diss**	**Diss/SD**	**Contrib%**	**Cum.%**	
otu3	0.03	29.32	14.65	1.73	20.26	20.26	*Cadophora*
otu1	31.6	11.49	10.33	1.96	14.28	34.54	*Mycosphaerella_tassiana*
otu4	0.01	12.1	6.04	1.84	8.36	42.9	Heliotales_sps.
otu5	6.48	4.06	3.21	1.55	4.44	47.34	Pleosporales_sps.
otu6	7.36	1.83	2.81	1.05	3.89	51.23	*Vishniacozyma victoriae*

Table S2: PERMANOVA results for bacterial communities in meadow fescue (*Festuca pratensis*). df: degrees of freedom, SS: Sum of squares, MS:Mean sum of squares, Pseudo-F: F-value by permutation, P(perm): p-values based on more than 900 permutations.

**Source**	**df**	**SS**	**MS**	**Pseudo-F**	**P(perm)**	**Unique perms**
**Tissue**	1	28791	28791	16.32	0.001	998
**Endophyte**	3	4460.3	1486.8	0.843	0.889	994
**Tissue x Endophyte**	3	4801.1	1600.4	0.907	0.75	999
